# Death and the artificial placenta

**DOI:** 10.1093/jlb/lsae013

**Published:** 2024-07-16

**Authors:** Anna Nelson, Elizabeth Chloe Romanis, Victoria Adkins, Christina Weis, Karolina Kuberska

**Affiliations:** School of Law, University of Sheffield, Sheffield, S3 7ND, UK; Durham Law School, Durham University, Durham, DH1 3LE, UK; Petrie-Flom Center for Health Law Policy, Biotechnology, and Bioethics, Harvard University, Cambridge, MA 02138, UK; School of Law and Criminology, University of Greenwich, London, SE10 9LS, UK; School of Allied Health Sciences, De Montfort University, Leicester, LE1 9BH, UK; Department of Public Health and Primary Care, University of Cambridge, Cambridge, CB2 0SR, UK

**Keywords:** artificial placenta, artificial womb, stillbirth, miscarriage, death

## Abstract

Artificial Amnion and Placenta Technology (AAPT)—sometimes referred to as ‘Artificial Womb Technology’—could provide an extracorporeal alternative to bodily gestations, allowing a fetus delivered prematurely from the human uterus to continue development while maintaining fetal physiology. As AAPT moves nearer to being used in humans, important ethical and legal questions remain unanswered. In this paper, we explore how the death of the entity sustained by AAPT would be characterized in law. This question matters, as legal ambiguity in this area has the potential to compound uncertainty and the suffering of newly bereaved parent(s). We first identify the existing criteria used to delineate the legal characterization of death, which occurs before birth or during the immediate neonatal period in England and Wales. We then demonstrate that attempting to apply these in the context of AAPT gives rise to a number of challenges, which make it impossible to reach a definitive conclusion as to the nature of death in AAPT using the current legal framework. In doing so, we demonstrate that the current legal framework in England and Wales may be unable to adequately capture the situation of an entity being sustained by AAPT.

## I. INTRODUCTION

Questions about the meaning of life and death give rise to thorny ethical debate. Unsurprisingly, these issues also remain legally contentious. Novel technologies in development, such as Artificial Amnion and Placenta Technology (AAPT) (also sometimes referred to as ‘artificial womb technology’ in literature), have the potential to complicate these questions even further. In this paper, we explore how the law might define death of an entity gestating in an artificial placenta device.

Theoretically, an AAPT device for humans would provide an environment that mimics the human placenta, allowing its subject (the ‘gestateling’)^1^ to maintain fetal physiology while being sustained by the device.^2^ The successful development of AAPT could therefore enable gestation outside the mammalian body, also known as ectogestation.^3^ In 2017, a team based at the Children’s Hospital in Philadelphia announced that their biobag device was used to successfully support lambs that had been delivered extremely prematurely by cesarean section at 110 days, considered the edge of viability for lambs.^4^ Since this initial success, research continues to bring the technology closer to clinical translation for humans^5^ and a number of other teams are also developing and testing their own devices with very conceptually similar designs.^6^ The potential for clinical translation for humans should be taken seriously: the EXTEND (EXTrauterine Environment for Neonatal Development) therapy device, created by the team at the Children’s Hospital in Philadelphia,^7^ has been designated as a ‘Breakthrough Device’ by the US Food and Drug Administration, as part of a program designed to expediate the development and regulatory review of medical devices to aid timely clinical translation of promising research.^8^ In 2022, EXTEND was described by the research team as nearing human use ^9^ (though it is worth noting that in 2023 the FDA’s Pediatric Advisory Committee indicated that they did not believe that it was appropriate for ‘first in human’ trials to begin).^10^

Since several of the research teams have expressed their intention to conduct trials in humans in the relatively near future, and/or have set out a roadmap for doing so, the pressing ethical issues in clinical translation are increasingly important. Though the bioethics literature on artificial placentas initially explored the idea of *complete* ectogestation, there is a growing body of work focusing on the specific issues raised by *partial* ectogestation.^11^ This is the use of the technology to take over gestation when a pregnancy cannot be sustained to term, or when a pregnant person does not want to sustain their pregnancy to term.^12^ Given current clinical developments, partial ectogestation is far closer to being used in humans.^13^ Such uses bring their own specific ethical issues. These involve questions about informed decision making in the context of novel technologies, the availability of the device within hospital wards—and equality of access to these devices, as well as the likely impacts of this unfamiliar technology upon parents and staff, amongst many others.^14^ Additionally, there is a growing body of literature that has considered the implications of partial ectogestation, for example, in relation to gender equality and the potential to bolster pregnant people’s ability to make choices about their bodies, and their experience of giving birth.^15^ However, there is much less literature on matters of clinical translation (with some notable exceptions),^16^ and even less reflecting on the potential ways partial ectogestation may be experienced by parents or healthcare professionals who will operate artificial placenta devices.^17^

As explored in detail in section (III), we argue that there is a conceptual distinction between a fetus and the entity being gestated in an AAPT device. While some may consider a fetus that has been extracted from the human womb and transferred to an APPT device as uncomplicatedly ‘born alive’, we posit that the matter is more complicated and, as Romanis has explored in detail elsewhere,^18^ it is not clear that the entity in the AAPT device would fall within the existing legal definition of ‘born alive’. Therefore, we argue that the existence of this new kind of entity requires us to interrogate existing legal understandings of life and death to assess whether, and how, they are able to accommodate the novel realities brought about by the development of this technology. The task of understanding how death of the entity gestating in the AAPT device would be interpreted in law is an important and urgent one, given the profound impact such an event can have.

This paper explores how the death of an entity being sustained by this device may be categorized legally, while recognizing that legal constructions of reproductive loss do not always provide an accurate reflection of how those who actually experience such loss characterize it. We focus specifically on the law in England and Wales in our analysis; however, we suggest that the considerations we highlight may also have relevance when thinking about this issue in other jurisdictional contexts. This has particular significance as current clinical research suggests that partial ectogestation, in its initial application, is likely to be utilized where a pregnancy becomes dangerous in the late second or third trimester or where a pregnant person goes into spontaneous premature labor. Similarly to the clinical translation of many other technologies, it is likely that the artificial placenta will not always be able to ensure the survival of the gestateling.

While matters relating to the cause of death may be both ethically and legally relevant (for example, in instances where it can be shown that a person is responsible through negligence),^19^ we do not address the issues related to responsibility or redress. Rather, our focus is on how the law may categorize such a death and whether current legal provisions can align with the experience of putative parents. This is of key importance given that the law on death in the context of pregnancy is already complex and subject to criticism. As a matter of law—in England and Wales—any intentional ending of a pregnancy (even where it is intended that the fetus will survive) is considered an unlawful procurement of miscarriage—an abortion.^20^ This means that in the circumstances we are describing, in which a gestating entity is removed from the pregnant body to be sustained by an artificial placenta, there has, legally, been an abortion.^21^ We acknowledge that in lay terms abortions are considered to encompass both the end of the pregnancy and the death of the fetus^22^ and so we believe that in circumstances in which there is fetal transfer to AAPT, the pregnant person is unlikely to consider themselves as having had an abortion. Because of this lay understanding and because abortion describes—in law—only the ending of the pregnancy, we do not discuss abortion further in this paper. The matter at hand is how we legally categorize the death of a gestating entity sustained by an artificial placenta (not how we categorize the ending of the pregnancy).

## II. ARTIFICIAL AMNION AND PLACENTA TECHNOLOGY

The development of AAPT to facilitate gestation outside of the body has long been imagined: 2023 marks the centenary of JBS Haldan’s *Daedalus* lecture at Cambridge, in which the prospect was first theorized.^23^ The devices in development today are not intended to replace full human pregnancies but to specifically solve some of the existing problems in neonatal intensive care,^24^ given that prematurity is the leading cause of child death worldwide.^25^ First, there are physiological limits on survival: neonates delivered at or before 22 weeks gestation are usually incapable of surviving in the external environment as they are physically unable to withstand the impact of gas ventilation.^26^ For neonates with more developed lungs, conventional neonatal intensive care has increased the chance of survival, however, with significant limitations. Morbidity and mortality result from complications of intensive care: the necessary invasive interventions are associated with high risks of infection^27^ and mechanical gas ventilation can cause damage to the underdeveloped lung tissue.^28^ Survival rates of preterms at the threshold of viability (22–25 weeks) have slightly increased with modern neonatal intensive care; however, there remains a high incidence of complications that are either life-limiting and/or have a serious impact on quality of life.^29^ Given the limitations of conventional therapies (the neonate needs to be capable of gas ventilation and of withstanding the associated risks of that ventilation), fetal scientists have sought to develop an alternative approach to support entities too premature to tolerate conventional neonatal intensive care treatment: a device to support continued gestation.^30^

All current prototype devices being tested in animals have similar objectives in their design. These models consist of a sealed system of warmed amniotic fluid in which the gestateling rests, a set of cannulas that are connected to the two arteries and one vein of an umbilical cord to deliver nutrients/remove waste from the system and to run the blood via a pump-less oxygenator.^31^ Crucially, the system allows the subject to obtain oxygen through *liquid-based* ventilation: meaning that the lungs can continue to develop in the same way as they would if gestation continued in a standard way.^32^ To prevent the lungs clearing when the fetus is removed from the uterus, it is temporarily paralyzed for transfer to the AAPT device.^33^

AAPT marks a radical paradigm shift in physiological approach:^34^ ‘the central principle underlying the iterative development of [artificial placentas] is to treat extremely preterm infants as fetuses, rather than as small babies’.^35^ Because the design of AAPT requires the gestateling to have reached a certain point of fetal development,^36^ this kind of device will never be capable of *complete* ectogestation.^37^ However, there is reason to think that this device will be able to sustain human entities delivered from bodily gestation earlier than those who can be supported in neonatal intensive care. At present, the threshold of neonatal viability (the gestational age at which entities would be provided with intensive care) is around 22–24 weeks for the reasons described above.^38^ AAPT, however, does not require any specific level of gestational maturity beyond having fetal physiology (attained at around 13 weeks), which means, in theory, it could be used to support entities far too gestationally immature for standard neonatal intensive care.^39^ This fact may intensify some of the complexities in the definition of an entity gestating *extra uterum* (the ‘gestateling’).^40^

As the science and scholarship around extracorporeal gestation develops, so too does the language. Though variances in terminology are perhaps inevitable at this stage, the fact that different authors may use different terminology to describe the same phenomenon or process can lead to uncertainty and confusion. In this paper, we use the term ‘Artificial Amnion and Placenta Technology’ (AAPT) to describe the technology that facilitates *ex utero* gestation, rather than the more common term ‘Artificial Womb’. There are two primary reasons for this. The first is to circumvent narratives of alternative: partial extracorporeal gestation does not *replace* human gestational work, but rather takes over the work of gestation at a certain stage in the pregnancy. By using language that clearly distinguishes *ex utero* gestation from the bodily work that precedes it, we can resist the notion that *in utero* and *ex utero* gestation, pregnant person, and machine, are interchangeable.^41^ The second relates to the symbolic nature of the word ‘womb’, which for some has connotations of maternal nurture and care-giving.^42^ By using more neutral and less emotive terms, we are able to create a space for exploring the technicalities of this phenomenon with more precise language.

## III. THE GESTATELING

There has been some debate amongst philosophers and bioethicists as to whether there is a difference between conventional neonatal intensive care and AAPT. Whether such a distinction exists is material in understanding what the subject of the artificial placenta is*.* If AAPT is understood as being merely an extension of conventional neonatal intensive care, the death of the entity within AAPT poses no new legal challenges—this would fit squarely within the existing understanding of neonatal death.

We take the position that AAPT is not merely another form of neonatal intensive care. This centers on the argument, first advanced by Romanis^43^ and further supported by Kingma and Finn,^44^ that there is a conceptual difference between conventional neonatal intensive care and an artificial placenta. The crux of the distinction these authors highlight frames an artificial placenta as ‘capable of continuing gestation – the process of human development that happens before birth – ex utero, as opposed to facilitating incubation’.^45^ There are, therefore, differences in physicality, physiology, and behavior that distinguish a gestateling from both a fetus and a neonate.^46^ The gestateling is distinct from a fetus because of its location: a fetus is a part of a pregnant person,^47^ whereas a gestateling is no longer a part of a pregnant person.^48^ However, a fetus and a gestateling share a number of similarities in that they have the same physiology^49^ (the artificial placenta is designed specifically to maintain fetal physiology)^50^ and thus behavioral capacities. These similarities a gestateling shares with a fetus are what distinguish it from a neonate and illustrate that while the fetus may be *birthed* by location, it is not completely *born*, in the sense that it has not made the necessary physiological adaptations to survive in the external environment.^51^ Neonates are human entities that have been both *birthed* (so no longer a part of the pregnant person) and *born* (having the necessary physiological adaptations to survive in the external environment). Table 1 illustrates the differences between all three entities.

**Table 1:** Differences between fetus, gestateling, and neonate

Condition / Entity	Birthed (no longer part of a pregnant person)	Born (physiologically adapted to the external environment)
Fetus	No	No
Gestateling	Yes	No
Neonate	Yes	Yes

We find this distinction convincing and adopt the term ‘gestateling’ in the paper, coined by Romanis to describe the subject of the artificial placenta, to highlight its unique status as the subject of extracorporeal gestation.^52^ The term ‘fetonate’ has been recently introduced by members of the Philadelphia study team to describe the subject of the artificial placenta (in an ethics paper published in 2021).^53^ Segers and Romanis have suggested that the difference in terminology likely results from contextual factors. Clinicians may be seeking to use a term that is ‘easily recognizable to putative parents who may need this technology, and particularly those who may be the first consenting experimental users’.^54^ The term gestateling was coined, however, with the objective of distinguishing this entity from both a fetus and a neonate in very clear terms.^55^ We use the term gestateling for clarity given that legal regulation of death is the focus of this paper.

The existence of this conceptual distinction has been questioned by some scholars.^56^ We do not have space to defend our objections to these arguments here, though aspects of the dispute become relevant in our discussion throughout the paper. We note, however, that even those who reject the categorization of the AAPT subject as a gestateling implicitly seem to accept *some* relevant distinction between this entity and both the fetus and a conventional neonate.^57^

One key difference refers to the gestateling lacking ‘natality’—that is, the quality of being in the world in a situated sense.^58^ This difference will be material in our analysis because it will affect a putative parent’s experiences of AAPT and death within it.^59^ While a gestateling will be of considerable importance to the people who intend to parent it once born (in a legal sense), it cannot be interacted with in the same way as neonates in neonatal intensive care. In the artificial placenta, the gestateling will not receive ‘situated care’, such as the touching and holding from caregivers.^60^ Instead, it will be gestated by the technology that will mediate the relevant human relationships.^61^ The gestateling’s physically inaccessible yet visually accessible existence is in stark contrast to the skin-to-skin contact practices that are encouraged with parent(s) in neonatal intensive care both to facilitate bonding (for the physical and mental health of the birthing person and/or intended parent)^62^ and to assist the neonate with physiological adaptations to the external environment (benefits include, for example, enhanced respiratory stability).^63^ The ‘having of a gestateling’ will be a novel experience; the offspring will be delivered and thus separated from the body that gestated it, while remaining physically inaccessible because it remains in the process of gestation facilitated by machine.

As a final point, none of the academics that have defended a distinction between gestatelings and neonates have claimed that gestatelings ought to be afforded no moral or legal protections. Acknowledging the differences between fetuses, gestatelings, and neonates is a matter of metaphysical accuracy, but also of recognizing and paying close attention to conceptual differences between entities that might impact on the experiences of people attempting to reproduce. There is a need for further interrogation of both the moral meaning and legal significance of the distinction between being birthed and being born—specifically, in terms of what legal protections might be afforded to the gestateling.^64^ There have been some suggestions that the gestateling occupies a sort of middle ground—it might be afforded more protections than a fetus, but maybe fewer than a neonate.^65^ However, this has not been fully fleshed out. We do not have the space to engage meaningfully in that conversation; instead, we focus on the application of the current law and the potential experiences of people who have social and professional relationships with the gestateling.

## IV. FACTORS DETERMINING DEATH STATUS IN AAPT

Generally, conversations about AAPT occur in the context of rescuing and sustaining life. However, precisely because it is being used to save life, it is important to recognize that some gestatelings will die within AAPT. The death of the gestateling does not straightforwardly fit within the existing legal framework. As established in the previous section, the gestateling is a novel entity, which means that its death is a novel event, requiring reexamination of existing ethical and legal understandings against which it is uniquely situated. It is not the case, however, that this new kind of death in AAPT problematizes an otherwise satisfactory legal status quo. Rather, we contend that the challenges associated with legally characterizing the death of the gestateling in AAPT further illuminate—and may exacerbate—pre-existing inadequacies with the law as it relates to pregnancy loss and early neonatal loss. We argue that untangling these issues is key as legal complexities have the potential to result in real human suffering, compounding the already difficult experience of undergoing premature birth as well as experiencing the loss of a pregnancy.

The legal characterization of pregnancy loss has important practical and symbolic implications.^66^ For example, some who lose their pregnancy before 24 weeks of gestational age find their exclusion from certain formal processes (such as registering their child and receiving a stillbirth certificate) unexpected and upsetting.^67^ Under employment law in England and Wales, maternity and paternity rights in relation to both pay and leave only, attach after 24 weeks or after live birth; meaning those who experience a miscarriage are excluded from these rights.^68^ These gaps between legal provisions and the expectations of people experiencing pregnancy loss are likely to become more pronounced in the cases of deaths in AAPT.

In this section of the paper, we consider how death in the AAPT would, and could, be legally understood. This consideration is important given that such death is likely to occur. Such death will also likely be emotionally taxing for the parents and the healthcare professionals. Avoiding the added burden of legal ambiguity and complexity as to the nature of that death is therefore an important aim.^69^

The question of how death of the gestateling being sustained by the AAPT device would be legally characterized is a deceptively simple one. It may even seem strange to pose the question of how ‘death in the artificial placenta’ would be categorized, plainly—it might be thought—it would be death. However, if we take a closer look at the law, we see that questions about death during, and immediately following, gestation facilitated by pregnancy are complex. Depending on the exact circumstances, ‘death’ will be legally categorized as one of three things: miscarriage, stillbirth, or neonatal death. There are three factors that are used to categorize a death at the beginning of life as either a miscarriage, stillbirth, or neonatal death: (i) gestational age, (ii) location, and (iii) signs of life. These factors sometimes work in tandem in determining the categorization of death; however, in some circumstances only one or two of the factors can be determinative. In this section, we explore what these factors are and how they are applied to determine the categorization of a death at the beginning of life. In doing so, we consider whether each of the factors can be straightforwardly applied to the use of artificial placentas. By highlighting the complexities that the use of artificial placenta presents for these factors, we illustrate that, using the existing law, it is hard to know how death in AAPT context would be conceptualized in relation to these factors, how each factor is impacted by our conceptualization of AAPT, and how this determines how a death in AAPT is categorized. With a goal of using language that accurately reflects the part of the human body that the artificial placenta replicates, throughout this paper, we refer to the gestateling gestating in the artificial placenta despite the fact that a fetus does not gestate *in* a human placenta. We do this to connote the way in which the gestateling will be contained within the artificial placenta device and inaccessible to the social world in the same way as a fetus is in the human body.

It is important to acknowledge that how a death is categorized has important legal and symbolic implications regarding if/how the death is registered and whether the formerly pregnant person (and any second parent) has access to statutory maternity rights. Following a neonatal death, parents are entitled to all maternity leave, paternity leave, and additionally bereavement leave (all compensated).^70^ In this case, both the birth and death must be registered and a birth and death certificate are issued.^71^ Parents of a stillborn baby, like parents who experience a neonatal death, are entitled to all statutory benefits including maternity, paternity, and parental bereavement leave and pay.^72^ However, the death is registered differently: there is no certificate of both birth and death, but a certificate of stillbirth.^73^ When a pregnant person experiences a miscarriage, however, there is no entitlement to statutory maternity/paternity leave or bereavement leave (compensated or uncompensated), nor is any official certificate registering the death issued.^74^ These distinctions can have a significant impact on the experience of people who experience loss at the beginning of life.^75^

In what follows, we examine in closer detail the three criteria used to make determinations about the death status of an entity (whether it is a neonatal death, a stillbirth, or a miscarriage). Ultimately, each of these criteria is dependent on the others in different ways and their relevance is determined by context. In what follows, whilst we attempt to discuss each criterion separately as far as possible to illustrate the complications of each, the interdependence of the criteria will be apparent.

### IV.A. Gestational Age

Gestational age plays a central role in determining how to characterize death that occurs during pregnancy. The law of England and Wales distinguishes between types of pregnancy loss on the basis of *when* the fetal death occurs during pregnancy. Death of a fetus *in utero* up to 23 weeks and 6 days is classified as miscarriage; death of a fetus from the first day of the completed 24th week of pregnancy is a stillbirth and requires formal registration where a certificate of stillbirth is issued.^76^ The 24 weeks +0 days point, thus, provides a clear distinction between miscarriage and stillbirth; with any loss up to 23 + 6 weeks falling within the former category. For those who experience pregnancy loss, this has significant implications regarding registration and statutory maternity rights.

The gestational age boundary, which the law has constructed between miscarriage and stillbirth, is founded upon scientific understandings of viability.^77^ In the law of England and Wales, viability refers to the capability to be ‘born alive’, and survive for ‘some time’ by breathing—rather than ‘being born alive and surviving in the longer term’.^78^ As medicine and technology have a tendency to develop faster than the law, this can cause tension as legal viability can become out of step with the scientific reality. The legal definition of stillbirth was last amended in 1992, when the Still Birth (Definition) Act 1992 reduced the stillbirth threshold from 28 to 24 weeks, to reflect scientific advances in neonatal care.^79^ In 1985, a working party set up by the Royal College of Obstetricians published a report ‘Fetal Viability and Clinical Practice’, which ‘noted significant progress in neonate survival rates and recommended that the age at which a foetus should be considered viable should be 24 weeks.’^80^ The point of viability has remained fixed at 24 weeks since then, although the British Association of Perinatal Medicine now recognizes 22 weeks as the point at which there is a potential for survival outside the womb,^81^ and the advent of AAPT has the potential to reduce this threshold further.^82^ Just as the scientific accuracy of the legal boundary can be questioned, so too can the clinical reality of ascertaining the gestational progress of any given pregnancy.^83^ There is also space to question whether the stillbirth/miscarriage boundary is, or ought to be, linked to viability at all, even if viability could be accurately defined and established.^84^ Though clearly imperfect, gestational age nonetheless is a key criterion in categorizing death that occurs during the course of a pregnancy, and this has important practical consequences.

In the absence of specific legislation determining the status of the gestateling in the artificial placenta, interpretation of existing legislation may use its gestational age as the criterion of determining the type of death. This criterion is relevant because, as we have noted, AAPT could facilitate the continued gestation of entities less gestationally mature than 24 weeks. A gestateling in the artificial placenta that dies before the 24 weeks +0 days of gestational age, subject to how the interplay of other criteria are determined, could be legally classified as a miscarriage and its existence would not be formally recorded. According to the same logic, death of a gestateling in the artificial placenta after the first day of the 24th week of gestational age could, again subject to how other criteria are also interpreted in context, be classified as stillbirth, requiring a certificate of stillbirth (but not separate birth and death certificates). The potential implications of gestational age, when considered alongside other factors, such as location, are explored in more depth below.

For now, it is clear that gestational age classifications could potentially be very much at odds with events observable to the parents and healthcare staff—the human entity that died was *ex utero* and subject to techno-medical process in AAPT prior to its death. Consequently, classifications relying on existing categories of pregnancy loss dependent on gestational age could exacerbate the suffering of parents experiencing a reproductive loss mediated via artificial placenta technology.

### IV.B. Location

For most people from Euro-American cultures, the baby exiting the vagina (or abdomen) is the physical manifestation of a new life. It is this change in location, from being *a part of* the pregnant individual’s body to being *no longer a part* of the pregnant individual’s body that marks its visible entrance into the world. During a pregnancy, it is generally understood that the developing human entity is a fetus, and this fetus becomes a baby once it has exited the birth-canal/abdomen. Fetus is a routinely used word, and even those people who use the language of ‘baby’ during a pregnancy to refer to their future child still see the exit of the ‘baby’ from the body as a transformative moment.^85^ This location change often signifies the culmination of gestation and marks the beginning of parenthood, which can be shared with others. While this predominant cultural account of birth does not use the term ‘location’ to describe what occurs during what is considered the momentous occasion at the beginning of life, it is nevertheless the location change of the fetus/baby from *part of* the pregnant individual’s body to *not part of* it that is central. Therefore, if an entity dies before this central moment, it is understood to be different, than if it dies *after* entering the world.

This lay understanding of pregnancy and childbirth is somewhat reflected in the law since a change in location maps onto what is also a legally transformative moment in the recognition of the developing human entity as a legal person. In *Paton v British Pregnancy Advisory Service*,^86^ Sir George Baker confirmed that:

[t]he foetus cannot, in English law … have a right of its own at least until it is born and has a separate existence from its mother.’^87^ The Supreme Court of Canada has similarly emphasized that ‘[t]he common law has always distinguished between an unborn child and a child after birth … For legal purposes there are great differences between the unborn and the born child.^88^

The significance of a change in location (from part of a pregnant person to an independent existence) is further crystalized in several statutes. Change in location is referred to in law as birth and defined as the moment at which a child ‘first has a life separate from his mother.’^89^ Similarly, the Infant Life Preservation Act 1929 refers to a birthed child as having an ‘existence independent of its mother’.^90^

Location is, in socio-cultural terms, therefore understood as an indicator of what an entity is. In legal terms, location change marks the recognition of a new legal person. Importantly, this indicator is a fixed binary in that a legal person either exists or does not. A fetus, which is a part of a pregnant person’s body, is not a legal person;^91^ whereas a baby, who exists external to the formerly pregnant person’s body,^92^ is a legal person. There is some case law that has considered whether an entity has legal personality (and thus is a baby rather than a fetus) as a matter of whether a location transition has occurred. These decisions emphasize that a birth occurs where a fetus has been completely expelled from the pregnant person’s body.^93^ As one example, in *R v Crutchley*, it was held that an entity is birthed when ‘the whole body of the child had come forth from the body of the mother’.^94^ These decisions—all reiterating that an entity must have a separate existence for legal personality—reinforce that an entity’s location as a part of the pregnant person, or as a separate entity, is binary; being partially birthed is simply still not being birthed. In several cases considering what constitutes separate existence (a complete location change), a rudimentary approach is taken: an entity is a fetus when it is part of a pregnant person’s (located within the uterus) body; once it has exited the birth canal/via the abdomen, the entity becomes a baby with legal personality. Location change is what is recognized. Thus, an entity that is *physically on the outside* of the pregnant person’s body, even if there are ways in which we might say the entity is still a part of the pregnant person, eg, if the umbilical cord is still attached, is legally birthed.^95^

The emergence of a human entity from a pregnant body (the location change)—as a legally and culturally significant event—has a long history and the justifications for ‘legal personality at birth’ have evolved around it—especially in the Euro-American world.^96^ English Courts have been clear that location change is legally relevant because while a fetus is a part of a pregnant person’s body, it cannot be afforded any recognition since that would cost the pregnant person *their* autonomy and liberty.^97^ In 1988, Balcombe LJ held that the legal protections of children cannot apply to fetuses ‘since an unborn child … has no existence independent of its mother, the only purpose of [recognising a fetus as a child that can be the subject to the wardship jurisdiction] … is to enable the mother’s actions to be controlled’.^98^ He is explicit that any order relating to the wellbeing of the fetus is a lifestyle restriction on a pregnant person and thus a huge imposition on the liberty of pregnant persons.^99^ The decision is focused on a narrow question and it places location at the center—the law thus places considerable emphasis on the location of developing human entities and this is important because of the impact that this has on the rights of pregnant persons.^100^

Although the law quite clearly denotes the concept of location as meaning being either in (as a part of) or outside of the pregnant person’s body, the use of artificial placentas may introduce a construction of location as being either in or outside of a uterine-like environment.^101^ The artificial placenta, in imitating the process of gestation that occurs within the human placenta, may be considered as a continuation or replication of that uterine environment. It may therefore be considered that the fetus has not changed location *per se* since it would still be within a uterine environment, albeit an artificial one. If this is the case, the death of the gestateling in an artificial placenta would be akin to the death of the fetus while it remains a part of the pregnant person. As has been set out above, a death that occurs inside of a pregnant person’s body is determined by the gestational age of the fetus. It may be presumed, therefore, that those gestational markers would simply be translated to the gestateling—ie, if the gestateling dies in an artificial placenta before 23 weeks +6 days gestation then its death would be classified as a miscarriage, whereas a death in the artificial placenta beyond this gestation would be considered a stillbirth. Table 2 illustrates the different potential definitions of death depending on how the artificial placenta is framed.

**Table 2:** Potential definitions of death dependent on framing of the artificial placenta

Interpretation of location/gestational age	*If* APPT is considered extension of uterus/human gestation	*If* extraction and transfer is considered birth
Extracted and transferred, and dies in AP before 23 + 6	Miscarriage	Neonatal death
Extracted and transferred before 23 + 6 but dies in AP after 24 + 0	Stillbirth	Neonatal death
Extracted and transferred after 23 + 6 and dies in AP after 24 + 0	Stillbirth	Neonatal death

However, the present law clearly links location to the pregnant body in its entirety, as opposed to merely focusing on the fetus’ position as an entity sustained by a placenta/uterus. A narrow focus on location as the only factor that matters in determining the status of a gestateling would not only undermine the larger role that the pregnant person’s body plays in sustaining gestation but would also cause legal dilemmas as to whether the fetus would have legal personality as it exits the uterus and moves down the birth canal. The current law is quite clear that it is the expulsion from the pregnant person’s *body* (and not from the placenta/uterus), however that expulsion takes place, that is relevant.

If the determinative factor of location were to be connected to the developing human entity being in or outside of a uterine-like environment, the legal status of fetuses and gestatelings would become analogous. If being within a uterine-like environment equated to having no legal personality, then a gestateling in an artificial placenta would have no legal rights, potentially exposing it to harms.^102^ This would also mean that its death within the artificial placenta would not be recognized in the same way as the death of a neonate (although it may be considered a miscarriage or stillbirth, depending on its gestational age). Alternatively, if it was determined that the gestateling should have its death recognized in the same way as a neonatal death, then there is the risk that the same argument could be made for the fetus that is a part of a human uterus, since it is also in a uterine-like environment. Recognizing a fetal death as akin to a neonatal death necessarily involves recognizing that the fetus has legal rights because the status of death is determined by the legal status of the entity that dies. In allocating fetal rights in this way, established precedent that operates to protect the bodily rights of the pregnant person is undermined. Since the current law does not allocate legal personhood to the fetus and this protects the rights and autonomy of pregnant persons, it seems unlikely that a narrow account of location that focuses purely on being in or outside of a uterine-like environment can determine legal status.

A further complexity arises in the short space of time in which the pregnancy has ended and the entity is no longer a part of the pregnant person but is not yet being supported by the AAPT device. There will be a period of time when the gestated entity, being no longer part of the pregnant person, seemingly might be thought to be ‘born’ because of the relevance of location (it is not part of the pregnant body and not sustained by another uterine-like environment). If the death of the entity were to occur during the short window that it is outside both the gestating human body and the artificial placenta device, some may argue that on the basis of location the death would legally amount to a neonatal death. However, within this short period, the physiology of the entity is maintained as fetal physiology (so that the entity can continue gestating once supported by the artificial placenta). The intersection between location and the ‘born alive’ rule becomes material.

In determining the status of an entity, the law is clear that an existence separate from a pregnant person’s body is a factor that carries significant weight. Further to this, the acquiring of legal status prescribes that the fetus be ‘born alive’, which is discussed in the next section. While it is difficult to consider the legal role of location in isolation, it is notable that to be born alive, the location change from inside to outside the pregnant person’s body must take place *first.* Thus, location is a prerequisite in the assessment of what an entity is, what its legal status is, and thus what its death is.

### IV.C. Signs of Life

Legally speaking, exit from the gestational body is not alone determinative of life. Only where an entity is born alive will it be afforded legal personhood, and gain the rights associated with this.^103^ There is no specific legal definition for what it means to be born alive. However, the statutory definition of stillbirth is instructive in determining when a child will be deemed, in the eyes of the law, to have been born alive. The Births and Deaths Registration Act 1953 establishes that a:

‘still-born child’ means a child which has issued forth from its mother after the twenty-fourth week of pregnancy and which did not at any time after being completely expelled from its mother breathe or show any other signs of life, and the expression ‘still-birth’ shall be construed accordingly.^104^

Therefore, the determination of life in the UK rests upon whether, *upon separation from the gestational mother*,^105^ the infant breathes *or* displays other signs of life.^106^

The law of England and Wales is not prescriptive on what amounts to signs of life sufficient to evidence that an infant has been born alive,^107^ relying instead on clinical guidance.^108^ Caution is needed when examining clinical guidance, as the term ‘signs of life’ is used to refer both to legally significant signs of life (those which occur following separation from the gestational mother) and other clinically important signs of life that can be detected *in utero,* but which do not themselves confer a particular legal status upon the fetus.^109^ In what remains of the discussion, ‘signs of life’ will be used to refer to legally significant signs of life displayed following separation from the pregnant person.

According to clinical guidance, for an infant to be deemed to have been born alive upon separation from the pregnant person, it must display one of the following: (1) easily visible heartbeat, (2) visible palpitation of the cord after it has been clamped, (3) breathing, crying, or sustained gasps, and/or (4) definite movement of arms or legs.^110^ Though signs of life need not persist for long, it is emphasized that ‘fleeting reflex activity observed only in the first minute after birth does not warrant classification as signs of life’.^111^ This requirement that activity be more than fleeting accords with academic assertions that for activities to be sufficient to indicate legally significant life, they must be more than primitive in nature.^112^

While clinical guidance appears to indicate that the existence of any of the activities in (1)–(4) would be sufficient to indicate life, there remains some debate in the legal sphere regarding whether it is possible to determine that an infant has been born alive in the absence of breathing. Older case law seems to indicate that an infant could only be determined to have been born alive if breathing occurred^113^ and as Romanis has noted ‘in modern coroners’ inquests determining whether a child was born alive, factual findings focus on whether there was post-birth breathing’.^114^ Were this focus on breathing to be maintained, then this would support the argument that the gestateling is *not born alive*, and consequently point toward the appropriate legal treatment of death in the AAPT device as a form of death before birth. This is because one of the most salient features of AAPT technology (as opposed to conventional NICU technology) is that it bypasses the respiratory system; it specifically does not require the gestateling to be able to sustain gas ventilation, instead facilitating a liquid-based ventilation.

However, some doubt was cast upon the absolute legal necessity of breathing by the judgment in the Australian case of *R v Iby.*^115^ The judge in *Iby* took a broader approach to the question of whether the born alive threshold had been satisfied, stating that ‘[t]here is no single test of what constitutes “life”’.^116^ This has been treated with approval in subsequent cases in other Australian states; for example, in the South Australian Supreme Court case *R v Barratt (No 3)*, Vanstone J stated that ‘any sign of life after birth would be sufficient’ to ‘prove that the child was born alive’.^117^ As these were Australian cases, they do not carry binding legal authority in the English court; however, they have persuasive weight. At present, the practical significance of this debate is limited, and it has been noted that the law in England is very clearly centered around breathing.^118^. It is here where we can see the significance of the debate surrounding whether breathing is one of a number of potentially determinative signs of life, or whether it is a necessity in establishing life. If UK law follows the Australian approach and broadens the approach to the born alive threshold beyond the necessity for breath, then the argument that the gestateling within AAPT displays legally relevant signs of life remains open.^119^

Nonetheless, despite the uncertainty regarding the exact nature of this criterion, it is clear that signs of life play a determinative role in establishing whether an infant is alive or not. As noted in the previous section, an infant that is delivered from the body at any gestational age and displays signs of life will be deemed born alive, it does not matter whether they have completed the 24th week of gestation.^120^ However, if there are no signs of life upon delivery—then gestational age remains the important factor in distinguishing between miscarriage and stillbirth.^121^

The prospect of AAPT poses an interesting challenge in relation to the application of the signs of life criteria to determine whether an entity is born alive.^122^ We know that the fetus must be paralyzed for transfer to the AAPT, by preventing it from drawing breath, oxygenation of blood can be continued without the involvement of underdeveloped lungs, and during this transfer, there will be a (short) period of time where the gestateling is outside of both the human uterus and the AAPT device, and visible to clinicians. What is the significance of the fact that the potential for signs of life is *deliberately suspended* during this time, through the administration of a paralyzing agent? Depending on the design of the AAPT, the gestateling within this may be—to some extent at least—visible to clinicians and parents (for instance via special cameras) in a way that the fetus *in utero* is not. It is plausible that one would be able to establish ‘definite movement of the arms and legs’, and perhaps even the existence of an easily visible heartbeat. Would these qualify as signs of life, signifying that the gestateling has been born alive and thus affording it legal personhood? Romanis notes that it is a plausible interpretation of English law that the gestateling does not satisfy the definition of born alive for the purposes of establishing legal personhood, since these signs of life are not exertive. This has led to some debate in the philosophical literature about what signs of life should be considered meaningful or not. Colgrove, relying on the World Health Organization definition of a live birth, argues that the gestateling exhibits signs of life once it has been birthed and thus must be born.^123^ He claims gestatelings ‘just are newborns by definition’ because they are a living independent entity.^124^ Both Romanis and Kingma dismiss his claims as reductive, noting that his position—which claiming it appreciates the nuances in birth/being born—fails to do exactly that^125^ as Colgrove has ‘conflated any sign of *biological* life as evidence of a meaningful (in a philosophical or legal sense) life or birth’.^126^

At present, the signs of life criterion does not become legally significant until a relevant change in location has occurred. If AAPT is (legally) akin to the *in utero* environment, then the fact that the gestateling is moving and has a heartbeat is not relevant for legal personality (just like moving and having a heartbeat are not sufficient criteria for conferring legal personhood for the fetus *in utero*). However, if the change of location from inside to outside of the pregnant person’s body is considered significant, then the question arises of how to apply the current signs of life criterion to the gestateling—given that this entity will physiologically diverge from a newborn in a number of ways. Similarly, if one considers the time (during transfer) outside of either the pregnant person’s body or the APPT device as significant, then challenging questions arise about how to accommodate signs of life criterion during this time are implicated.

If either of the latter two positions are taken, a challenge could be raised around whether it is possible, or indeed desirable, to attempt to apply the existing signs of life criterion to this novel context at all. The determination of life on the basis of this criterion does not represent immutable fact; rather, it provides a clinically (and perhaps sociably) acceptable statement that sufficient evidence upon which a determination to this end can be made. Given the lack of a clear legal statement regarding what exactly amounts to signs of life, it may be more straightforward to simply create a new set of guidelines to deal specifically with determining the boundary between life and death in those instances where the entity delivered from the human womb continues gestation *ex utero.* Indeed, some may argue that as the *status quo* already lacks clarity, and is subject to existing debate,^127^ there is little value in attempting to apply the existing signs of life criterion to this novel situation as this will not likely facilitate legal clarity.

#### 1. Using these Factors to Characterize Death in the AAPT

From the point of view of the parents, and especially in the longer term, the (formal) recognition of the loss is likely to matter the most, so the use of the category of miscarriage may likely prove fundamentally misaligned with parents’ lived experience. If the death of the gestateling whose gestational age does not exceed 23 weeks and 6 days is classified as a miscarriage, there will not be a requirement of registration, statutory bereavement leave, or any other entitlements accompanying stillbirth and neonatal death. More importantly, the lack of a formal recognition of miscarriage will significantly clash with the observable—if failed—efforts of a team of healthcare professionals and the use of expensive, complex technology to preserve the life of a gestateling. If the death of such a gestateling is classified as a stillbirth *ipso facto*, it will mean that the criterion of gestational age loses its relevance in the context of the artificial placenta. If the death of such a gestateling is classified as a neonatal death, it will mean that criteria for signs of life in live birth have not included breathing on their own, putting into question decades of medical determinations of miscarriages. This illustrates how complex the matter is.

In attempting to apply the existing legal criteria speculatively to death that occurs during the use of AAPT, three core issues emerge about the application of these criteria, which make it impossible to reach a definitive conclusion as to the nature of death in AAPT using the current legal framework. These questions, at their root, are all related to the fundamental matter of how AAPT is understood: whether it is recognized as a conceptually distinct technology sustaining a novel human entity, or not.

Following our analysis of these factors and their possible interpretation, we are left with the following issues:

1. The relevance of gestational age becomes unclear, as it is dependent upon how AAPT is characterized (as an extension of NICU or as something novel).

2. The meaning of location may be altered by the introduction of AAPT, depending on how the technology is characterized.

3. The application of signs of life to AAPT is dependent on its characterization; it is also unclear, which signs of life are relevant.

The complex reality is that at different stages, different combinations of time, location, and signs of life are used to make legal determinations about whether an entity is alive and, if not, how its death should be legally characterized. As we have shown, how, when, and if each of the three criteria apply to AAPT remains dependent on how AAPT itself is constructed. For example, the relevance of gestational age when it comes to death in AAPT is dependent on whether the artificial placenta is considered an extension of human gestation and therefore akin to the human uterus or whether birth is considered to have taken place and as such gestational age no longer applies. Similarly, whilst location for current legal purposes relates to being in or outside of the gestator’s body, the AAPT presents the possibility of location being considered as in or outside of a uterine-like environment. How and whether this type of location matters depends on how the artificial placenta is conceptualized (ie, as akin to the human uterus or a system that functions post-birth). Further to this, signs of life will only be a relevant criterion regarding death in the AAPT if the location change of leaving the gestator’s body *is* considered birth and therefore it needs to be determined whether the entity is born alive before it enters the artificial placenta.

As well as the interdependence of the existing criteria, the introduction of AAPT also gives rise to questions about potential new meanings of each of the existing criteria—and to the possibility that new criteria may become significant.

The inability to coherently apply the existing relevant legal criteria to a death in AAPT suggests that significant focus needs to be placed on clarifying the legal position of such a death. The lack of clarity as to how a death in AAPT will be defined has the potential to cause suffering to the parents, as confusion about the nature of the event may impact upon their ability to process it. In the next section, we explore further how lived experience becomes central to the importance of determining how death in the artificially placenta is legally defined.

## V. IMPLICATIONS AND LIVED EXPERIENCE: ONE SIZE DOES NOT FIT ALL

Research carried out with parents who experienced loss at the margins between miscarriage/stillbirth and stillbirth/neonatal death has emphasized the important role that the appropriate terminology used by healthcare professionals’ plays in preparing parents for the physical and emotional realities of losing a baby.^128^ This, in turn, had a positive impact in terms of their longer term experiences of grief.^129^ This adds weight to our argument that careful consideration of the terminology used to talk about the death of the gestateling is important to avoid unnecessarily compounding the suffering of bereaved parents.

Research about parental experiences illustrates that even if legal clarity is achieved, it remains vital to recognize that parents may understand and experience the death of the gestateling in different ways regardless of how the law characterizes these deaths. For some, the legal definitions may map on their experience, for others the two will diverge. Lived experience is inevitably messier and more complex than the bright lines the law constructs. This is reflected by decades of interdisciplinary research into the significance of miscarriage to people who experienced it, which confirms that pre-24-week pregnancy loss can be accompanied by bereavement similar to that after stillbirth or neonatal death.^130^

Healthcare professionals and others involved in caring for the families that experienced pregnancy or baby loss have an important role to play in mediating the boundary between legal (and clinical) technicalities and personal realities. For example, the language used to discuss the death of a gestateling can be modified to reflect the beliefs and preferences of parents in the clinical setting—even if this diverges from the legal characterization thereof. The language of law is explicitly technical, terms must be carefully crafted to construct precise boundaries to guide individuals as to how to act in particular scenarios and to inform them of the consequences when such boundaries are crossed. Lived reality is, however, far more heterogeneous and nuanced, particularly in highly emotionally charged scenarios such as birth and death. Language that is appropriate for one person may feel disrespectful to another. Death of a gestateling in AAPT will likely require that healthcare professionals navigate an even more complex situation in those situations where this technology, which offers unprecedented hope for babies, fails.

Strict adherence to the language of the law in direct patient–doctor encounters ought not to be promoted. Rather, clinicians should aim to reflect the language of their individual patients and their families as far as possible—while ensuring they sensitively communicate the legal realities and practicalities of the situation. This will be complex, particularly in those instances where parents characterize the situation very differently than the law, which may exacerbate suffering—as we have seen in the context of miscarriage and stillbirth. Quality guidance and a real focus on communication in both education and continuing professional development will be necessary to help navigate this. A clear legal position on how death in AAPT is characterized would enable healthcare professionals to guide and care for parents experiencing this loss.

## VI. CONCLUSION

Death of the gestateling in the artificial placenta, similarly to death of the fetus in pregnancy or death of a neonate, may cause bereavement, irrespective of whether the legal status of this death is legally recognized as miscarriage, stillbirth, neonatal death, or a new category. At the same time, the legal determination of this death status may exacerbate the parents’ suffering, especially if this determination of the legal status does not match with the parents’ perceptions of what happened to what they may already understand as their child.^131^ Evidence from research on pregnancy loss has shown time and time again that the legal and clinical categories are often translated into language that bereaved parents find more appropriate and sensitive—even at the cost of generating some degree of confusion.^132^ This may likely be the situation of the gestateling in the AAPT, where new legal categories may not align with parental experience. This matters for a number of reasons—from having the language, social script, and a (legal) frame of reference for processing an experience of bereavement, to an opportunity for official recognition of the loss via the registration requirement, to statutory entitlements such as parental/bereavement leave,^133^ among others.

It is currently unclear how the law will determine the status of death in AAPT. In the absence of new legal categories for deaths in artificial placenta, existing categories of miscarriage, stillbirth, and neonatal death will be used—although it is not yet clear what criteria will be prioritized in the determination. We argued in this paper that the criteria used for current types of reproductive losses—gestational age, location, and signs of life—cannot be straightforwardly applied to the death of a gestateling. Grappling with the legal categorization of death of the gestateling *before* AAPT becomes a clinical reality is necessary because this allows specific procedures to be established to deal with this kind of death. If we wait until the situation arises, and then try to retrofit existing (flawed) law onto this novel situation, we will risk compounding the stress and emotional trauma of the newly bereaved parents.

Kranzberg stated that ‘Technology is neither good nor bad. Nor is it neutral’.^134^ Although medical technologies are usually motivated by the desire to improve health and healthcare, it is important to critically consider their broader effects on the society in which they are used. With the clinical application of the artificial placenta and amnion technology very much on the horizon, we should seek to understand whether there is space in the current legal framework to fully capture the consequences of this technology, both positive and negative. The current shape of the law fails to comprehensively address the circumstances that this new technology will make possible. This could be reflective of the fact that issues related to reproductive loss are rarely considered as important. Miscarriage and stillbirth have gained increasing visibility as they become less taboo to discuss and regulators have embraced the need to acknowledge reproductive loss and its significance.^135^ With the prospect of AAPT on the horizon, we have the opportunity to prevent the emotional suffering that legal uncertainty may cause and embed an understanding of loss in the technology’s regulation from the outset.

## Footnotes

1 We will use the term ‘gestateling’ to refer to the entity being gestated *ex utero.* Elizabeth Chloe Romanis, *Artificial Womb Technology and the Frontiers of Human Reproduction: Conceptual Differences and Potential Implications*, 44 J. Med. Ethics 751, 753 (2018).

2 Elselijn Kingma and Suki Finn, *Neonatal Incubator or Artificial Womb? Distinguishing Ectogestation and Ectogenesis using the Metaphysics of Pregnancy*, 34 Bioethics 254, 256 (2020); Elizabeth Chloe Romanis, *Challenging the ‘Born Alive’ Threshold: Fetal Surgery, Artificial Wombs, and the English Approach to Legal Personhood*, 28 Med. Law. Rev 93, 110–113 (2020); Elizabeth Chloe Romanis, *Artificial Womb Technology and the Significance of Birth: Why Gestatelings Are Not Newborns (or Fetuses)*, 45 J. Med. Ethics 728 (2019).

3 Kingma and Finn, *id.*

4 Jennifer Couzin-Frankel, *Fluid-Filled ‘Biobag’ Allows Premature Lambs to Develop Outside the Womb,*Science, April 25, 2017, https://www.science.org/content/article/fluid-filled-biobag-allows-premature-lambs-develop-outside-womb (accessed Sept. 19, 2023); Emily Partridge et al., *An Extra-uterine System to Physiologically Support the Extreme Premature Lamb*, 8 Nat. Commun. 15112, 2 (2017).

5 Ahmed El-Sabbagh et al., *Cerebral Oxygenation of Premature Lungs Supported by an Artificial Placent*a, 64 Asaio J. 552 (2018); Joseph Church et al., *Effects of an Artificial Placenta on Brain Development and Injury in Premature Lambs*, 53 jj. Pediatr. Surg. 1234 (2018); Patrick McGovern et al., *Neurologic Outcomes of the Premature Lamb in an Extrauterine Environment for Neonatal Development*, 55 J. Pediatr. Surg. 2115 (2020).

6 E. Joanne Verweij et al., *Ethical Development of Artificial Amniotic Sac and Placenta Technology: A Roadmap*, 9 Front. Pediatr. 793308 (2021); Harou Usuda et al. *Successful Use of an Artificial Placenta to Support Extremely Preterm Ovine Fetuses at the Border of Viability*, 221 Am. J. Obstet. Gynecol. 1 (2019); Alex Charest-Pekeski et al., *Achieving Sustained Extrauterine Life: Challenges of an Artificial Placenta in Fetal Pigs as a Model of the Preterm Human Fetus*, 9 Physiol. Rep. e14742 (2021).

7 Emily Partridge et al., *An EXTrauterine Environment for Neonatal Development: Extending Fetal Physiology Beyond The Womb*, 22 Semin. Fetal. Neonatal. Med. 404, 404 (2017).

8 US Food and Drug Administration, *Breakthrough Devices Program: Guidance for Industry and Food and Drug Administration Staff*, 2, https://www.fda.gov/media/108135/download?attachment (accessed Sept. 5, 2023).

9 Abby Larson et al., *The EXTrauterine Environment for Neonatal Development: Present and Future*, 25 Pediatr. Dev. Pathol. 253, 260 (2022).

10 Rachael Robertson, *Fda Advisors Grapple with Logistical, Ethical Issues of Artificial Womb Technology*, https://www.medpagetoday.com/obgyn/pregnancy/106426 (accessed Apr. 4, 2024).

11 The focus on partial ectogestation as a distinct process in need of ethical scrutiny was articulated in 2018, not long after the report of the biobag: see Romanis, *supra* note 1.

12 We use the term pregnant person throughout this paper both in recognition of the fact that not all those who gestate are women and because, conceptually, we argue that there is an important distinction between mothering (as a form of parenting) and gestating. On this second point, see Zaina Mahmoud and Elizabeth Chloe Romanis, *On Gestation and Motherhood*, 31 Med. Law. Rev. 109 (2023). Where we do use the term ‘gestational mother’ in the paper, we do so because this is a legal term of art—though we remain critical of the decision in *(McConnell and YY) v Registrar General* [2020] 2 All Er 813.

13 Larson et al., *supra* note 9.

14 Elizabeth Chloe Romanis, *Partial Ectogenesis: Freedom, Equality and Political Perspective*, 46 J. Med. Ethics. 89 (2020a); Elizabeth Chloe Romanis and Claire Horn, *Artificial Wombs and the Ectogenesis Conversation: A Misplaced Focus? Technology, Abortion, and Reproductive Freedom*, 13 Ijfab 174, 177 (2020); Elizabeth Chloe Romanis, Regulating the ‘Brave New World:’ Ethico-Legal Implications of the Quest for Partial Ectogenesis 28–35 (2020b) (PhD Thesis, University of Manchester).

15 Elizabeth Chloe Romanis, *Artificial Womb Technology and the Choice to Gestate Ex Utero: Is Partial Ectogenesis the Business of the Criminal Law?*, 28 Med. Law. Rev. 342 (2020c); Anna Nelson, *Should Delivery by Partial Ectogenesis Be Available on Request of the Pregnant Person*?, 15 Ijfab 1 (2022).

16 Elizabeth Chloe Romanis, *Artificial Womb Technology and Clinical Translation: Innovative Treatment or Medical Research?*, 34 Bioethics 392 (2019); Seppe Segers, *The Path Toward Ectogenesis: Looking Beyond the Technical Challenges*, 22 Bmc Med. Ethics. 1 (2021); Seppe Segers and Elizabeth Chloe Romanis, *Ethical, Translational, and Legal Issues Surrounding the Novel Adoption of Ectogestative Technologies*, 15 Risk. Manag. Healthc. Policy. 2207 (2022).

17 For example Elizabeth Chloe Romanis and Victoria Adkins, *Artificial Placentas, Pregnancy Loss and Loss-Sensitive Care*, J. Med. Ethics Online First: doi: 10.1136/jme-2023-109,412 (2023); Victoria Adkins, *The Impact of Ectogenesis on the Medicalisation of Pregnancy and Childbirth*, 47 J. Med. Ethics 239 (2020) and Victoria Adkins, The Birds and the Bees…and the Artificial Womb: Healthcare professionals views on the use and applicability of partial ectogenesis (2024) (PhD thesis, Royal Holloway, University of London).

18 Romanis (2018), *supra* note 1.

19 Jessica Schultz, *Development of Ectogenesis: How Will Artificial Wombs Affect the Legal Status of a Fetus or Embryo?*, 84 Chi-Kent. L. Rev. 877, 897 (2010).

20 Offenses Against the Person Act 1861, s.58—see Romanis (2020c), *supra* note 16. There is an interesting distinction between the definition of miscarriage in criminal law (defined as the ending of a pregnancy irrespective of whether there is fetal death) and miscarriage in civil law for the purposes of registration in which miscarriage is specifically defined to be a fetal death pre-24 weeks of pregnancy.

21 Romanis, *id*.

22 In contrast, the use of AAPT denotes an instance in which a pregnancy is ended but gestation is not—for further discussion of this distinction and its meaning in the context of procreative loss see: Elizabeth Chloe Romanis and Victoria Adkins, *Artificial Placentas, Pregnancy Loss and Loss-Sensitive Care*, 50 J. Med. Ethics 299, 307 (2024) 10.1136/jme-2023-109412

23 Rosemarie Tong, *Out of Body Gestation: In Whose Best Interests*?, in Ectogenesis: Artificial Womb Technology and the Future of Human Reproduction 60 (Scott Gelfand and John Shook eds., 2006).

24 For an ethical analysis see: Romanis, *supra* note 1.

25 Eric O. Ohuma et al*, National, Regional, and Global Estimates of Preterm Birth in 2020, With Trends from 2010: A Systematic Analysis*, 402 Lancet 1216 (2023)—published correction appears in Department of Error, Lancet 403 618 (2024); Joy Lawn and Mary Kinney, *Preterm Birth: Now the Leading Cause of Child Death Worldwide*, 6 Sci. Transl. Med. 263 (2014).

26 Couzin-Frankel, *supra* note 4.

27 Michael Brady, *Health-Care Associated Infections in the Neonatal Intensive Care Unit*, 33 Am. J. Infect. Control. 268 (2005).

28 Mohammed Ali Attar and Steven Donn, *Mechanisms of Ventilator-Induced Lung Injury in Premature Infants*, 7 Semin. Fetal. Neonatal. Med. 353 (2002).

29 Kate Costeloe et al., *Short Term Outcomes After Extreme Preterm Birth in England: Comparison of Two Birth Cohorts in 1995 and 2006 (the EPICure Studies*), 345 Bmj e7976 (2012); Andrei S. Morgan et al., *Birth Outcomes between 22 and 26 Weeks’ Gestation in National Population-Based Cohorts from Sweden, England and France*, 111 Acta. Paediatr. 59 (2022).

30 Brian Fallon and George Mychaliska, *Development of an Artificial Placenta for Support of Premature Infants: Narrative Review of the History, Recent Milestones, and Future Innovation*, 10 Transl. Pediatr. 1470, 1472 (2021).

31 Emily Partridge and Alan Flake, *The Artificial Womb, in*Fetal Therapy: Scientific Basis and Critical Appraisal of Clinical Benefits 83 (M. Kilby et al. eds., 2020).

32 Patrick McGovern et al., *Neurologic Outcomes of the Premature Lamb in an Extrauterine Environment for Neonatal Developmen*t, 55 J. Pediatr. Surg. 2115, 2122 (2020).

33 Personal communication.

34 Fallon and Mychaliska, *supra* note 30, at 1472.

35 Usuda et al., *supra* note 6.

36 There are two limitations that mean the gestateling must have reached a certain point of development. First, the gestateling must have fetal physiology necessarily because of features of the design. Second, the gestateling must be large enough for the smallest cannula designed for use with the device.

37 This said, if there ever were a device capable of complete ectogestation, some of our observations about defining death would still be relevant, and the question of what the death of a gestateling completely gestated ex utero would be even more perplexing.

38 NHS England, *Service Specifications—Neonatal Critical Care*, https://www.england.nhs.uk/commissioning/wp-content/uploads/sites/12/2015/01/e08-serv-spec-neonatal-critical.pdf (accessed Sept. 12, 2023).

39 For example, while recognizing that none of the current ‘clinical or experimental extra-uterine life support technologies’ are aiming for intervention pre-22 weeks gestational age, De Bie et al. recognize that were this technology to prove successful in relation to those delivered from the human womb >22 weeks, the technology could ‘conceivably’ be adapted for use at an earlier stage in the pregnancy: Felix De Bie et al., *Ethics Considerations Regarding Artificial Womb Technology for the Fetonate Ethics Considerations Regarding Artificial Womb Technology for the Fetonate*,23 Am. J. Bioeth. 67, 69 (2023).

40 Romanis, *supra* note 1.

41 Elizabeth Chloe Romanis et al., *Reviewing the Womb*, 47 J. Med. Ethics 820 (2021).

42 Ruth Landau, *Artificial Womb Versus Natural Birth: An Exploratory Study of Women’s Views*, 25 J. Reprod. Infant. Psychol. 4, 7 (2007).

43 Romanis, *supra* note 1; Romanis (2019), *supra* note 2; Romanis (2020), *supra* note 2; Romanis (2020b), *supra* note 15.

44 Kingma and Finn, *supra* note 2.

45 Romanis (2020b), *supra* note 15, at 103.

46 Kingma and Finn, *supra* note 2; Romanis (2019), *supra* note 2; Romanis (2020b), *supra* note 15.

47 Elselijn Kingma, *Were You a Part of Your Mother?*, 128 Mind 609 (2019).

48 Kingma and Finn, *supra* note 2, at 360.

49 Kingma and Finn, *supra* note 2; Romanis (2019), *supra* note 2.

50 Partridge and Flake, *supra* note 31.

51 Kingma and Finn, *supra* note 2; Romanis (2019), *supra* note 2; Romanis (2020), *supra* note 2.

52 Romanis, *supra* note 1, at 753.

53 Felix de Bie and others, A*rtificial Placenta and womb technology: Past, current, and future challenges towards clinical translation*, 41 Prenat. Diagn. 145 (2021).

54 Segers and Romanis, *supra* note 17, 2213.

55 Romanis (2020b), *supra* note 15, at 217.

56 Philip Wozniak, *Clinical Challenges to the Concept of Ectogestation*, 49 J. Med. Ethics 115 (2023); Nick Colgrove, *Subjects of Ectogenesis: Are ‘Gestatelings’ Fetuses, Newborns or Neither?*, 45 J. Med. Ethics 723, 224 (2019); Nick Colgrove, *Artificial Wombs, Birth and ‘Birth’: A Response to Romanis*, 46 J. Med. Ethics 554 (2020).

57 Elizabeth Chloe Romanis, *The Ethical and Legal Status of ‘Fetonates’ Or ‘Gestatelings’*, (2023) 23 Am. J. Bioeth. 90, 90 (2023).

58
Alison Stone, Being Born: Birth and Philosophy 3 (2019).

59 To understand parental views of AAPT, two of the authors are undertaking empirical research with parents who have had children cared for in neonatal intensive care. See https://wp.lancs.ac.uk/futureofhumanreproduction/sg-chloe-romanis/ for more information.

60 Romanis (2020b), *supra* note 15, at 205.

61 Romanis (2020b), *id.*

62 Hannah Jones and Nick Santamaria, *Physiological Benefits to Parents from Undertaking Skin-to-Skin Contact with their Neonate, in a Neonatal Intensive Special Care Unit*, 32 Scand. J. Caring. Sci. 1012 (2017); Joy Browne*, Early Relationship Environments: Physiology Off Skin-to-Skin Contact for Parents and their Preterm Infants*, 31 Clin. Perinatol. 287, 290 (2004).

63 Browne*, id.*

64 Kathrin Föhe, Siegfried Kropf and Stefan Avenarius, *Skin-to-Skin Contact Improves Gas Exchange in Premature Infants*, 20 J. Perinatol. 311 (2000).

65 Romanis (2020b), *supra* note 15, at 208; Romanis *supra* note 57, at 92.

66 Karolina Kuberska et al., *Death Before Birth: Liminal Bodies and Legal Frameworks*, in A Jurisprudence of the Body (Chris Dietz, Mitchell Travis and Micheal Thomson eds., 2020).

67 Aimee L. Middlemiss, *Pregnancy Remains, Infant Remains, or the Corpse of a Child? The Incoherent Governance of the Dead Foetal Body in England*, 26 Mortality. 299 (2021); Aimee L. Middlemiss and Susie Kilshaw, *Further Hierarchies of Loss: Tracking Relationality in Pregnancy Loss Experiences*, Omega (2023): doi.org/10.1177/00302228231182273.

68 The Maternity and Parental Leave etc. Regulations 1999; Maternity Action, *Miscarriage, Stillbirth and Neonatal Death—Rights to Time Off and Pay,*https://maternityaction.org.uk/advice/miscarriage-stillbirth-and-neonatal-death-rights-to-time-off-and-pay-for-parents/ (accessed Sept. 14, 2023); Aimee L. Middlemiss et al., *Employment Leave for Early Pregnancy Endings: A Biopolitical Reproductive Governance Analysis in England and Wales*, 31 Gender, Work & Organization 75 (2021).

69 There are also important questions to be answered regarding legal liability for death arising from human error, however, answering these lie outside of the scope of this paper.

70 The Parental Bereavement Leave Regulations 2020, Regulation 4.

71 Sands UK, *Pregnancy Loss and the Death of a Baby: Guidelines for Professionals (4th edition, 2016)*, https://www.sands.org.uk/professionals/sands-guidelines-4th-edition (accessed Sep. 8, 2023).

72 Gov.uk, *What to Do if a Child or Baby Dies*, https://www.gov.uk/after-a-death/if-a-child-or-baby-dies?step-by-step-nav=4f1fe77d-f43b-4581-baf9-e2600e2a2b7a (accessed Sep. 8, 2023).

73 Sands UK, *supra* note 71.

74 Karolina Kuberska and Sheelagh McGuinness, *Ambivalent Parallels in Registration and Certification of (Live) Birth, Stillbirth, and Miscarriage*, in Routledge Handbook of Law and Death (Marc Trabsky and Imogen Jones eds., forthcoming).

75 We return to this in more detail later in this paper.

76 Catherine Fairburn, *House of Commons Briefing Paper (2018): ‘Registration of Stillbirth’*, https://www.parliament.uk/globalassets/documents/commons-library/Registration-of-stillbirth-SN05595.pdf (accessed Apr. 12, 2023).

77 This is clear from parliamentary discussion: David Hanson and Ben Gummer, *Perinatal Mortality: Question for Department of Health [6 PQ 29604]* (11 March 2016), https://questions-statements.parliament.uk/written-questions/detail/2016-03-03/29604 (accessed Apr. 12, 2023); *Id.*

78 Elizabeth Chloe Romanis, *Is ‘Viability’ Viable? Abortion, Conceptual Confusion and the Law in England and Wales and the United States*, 7 J. Law. Biosci. 1 (2020d).

79 Still Birth (Definition) Act 1992; Similar amendments were made in the abortion context in 1990, where the upper gestational age limit for abortion under the Abortion Act 1967 was reduced from 28 to 24 weeks in recognition of advances in neonatal care—House of Commons Science and Technology Committee, *Scientific Developments Relating to the Abortion Act 1967 Twelfth Report of Session 2006–07* (hc 1045-I).

80 House of Commons Science and Technology Committee, *id.* at 21.

81 British Association of Perinatal Medicine, *Perinatal Management of Extreme Preterm Birth before 27 weeks of gestation A Framework for Practice (2019)*, https://hubble-live-assets.s3.eu-west-1.amazonaws.com/bapm/file_asset/file/30/Extreme_Preterm_28-11-19_FINAL.pdf (accessed Sept. 17, 2023).

82 Romanis (2020d), *supra* note 78. Note, that given the likely limitations on access to AAPT (at least initially), this technology will not directly reduce viability for the majority of fetuses. However, this does not mean the *potential* for fetuses to survive at a lower gestational age (regardless of how many actually have access to the technology which allows this) will not have clinical, legal or policy-based implications.

83 It is useful to note that gestational age, calculated from the first day of the last period, is a social and medical construct. People who menstruate have cycles of varying duration, pregnancy can only occur after ovulation, which can occur at a different day for different people and can vary from cycle to cycle. As a result, there is likely no pregnancy during the first 2 to 3 weeks of the period referred to as gestational age: Linda A. Hunter, *Issues in Pregnancy Dating: Revisiting the Evidence*, 54 J.M.W.H. 184 (2009).

84 Leah Eades, *Social Realities, Biological Realities: The 24-Week Foetus in Contemporary English Abortion Activism*, 74 Women’s Stud. Int. Forum. 20, 21 (2019); Romanis (2020d), *supra* note 78.

85 Suki Finn et al., *Pregnancy: Transformations in Philosophy and Legal Practice*, in Rewriting the History of Philosophy: Transformations (G. Anthony Bruno and Justin Vlasits eds., forthcoming).

86
*Paton v British Pregnancy Advisory Service Trustees* [1979] qb 276.

87
*Id*. at 279.

88
*Winnipeg Child and Family Services (Northwest Area) v. G. (D.F.)* [1997] 3 Scr 925, at [944].

89 Congenital Disabilities (Civil Liability) Act 1976, s.4(2)(a).

90 Infant Life Preservation Act 1929, s.1(1).

91
*Paton, supra* note 86; *Attorney-General’s Reference (No 3 of 1994)* [1997] 3 Wlr 421.

92 We acknowledge that a born baby is still physically dependent on caregivers and for people who breastfeed or chestfeed, the baby may at times still be thought of as part of their body. Nevertheless, the entity is not a physical part of their internal structure.

93
*R v Crutchey* [1837] 7 Car & P 814; *R v Poulton* [1832] 5 Car & P 328; *Rance and Another v Mid-Downs Health Authority and Another* [1991] 1 qb 587—For analysis see Romanis (2020), *supra* note 2, at 101–102.

94
*Crutchley*, *id*. per Parks B at [816].

95 On the irrelevance of umbilical attachment after birth canal exit see *Crutchley*, *id*; *R v Reeves* [1839] 9 Car & P 25; *R v Trilloe* [1842] Car & M 650; Romanis (2020), *supra* note 2, at 106.

96 See: Gerard Casey, *Pregnant Woman and Unborn Child: Legal Adversaries*?, 8 M.L.J.I. 75 (2002).

97 See the body of case law determining that a pregnant person is entitled to decline medical intervention, regardless of the impact this may have on the fetus: *St George’s Healthcare NHS Trust v. S* [1998] 3 wlr 936; *Re MB (An adult: medical treatment*) [1997] Ewca Civ 3093; *Tameside and Glossop Acute Services Trust v Ch (A Patient)* [1996] 1 Fcr 753; *Royal Free NHS Foundation Trust v. AB* [2014] Ewcop 50; *Re AA (Compulsorily Detained Patient: Elective Caesarean)* [2012] Ewhc 4378.

98
*Re F (In Utero)* [1988] Fam 22, per May lj at [143].

99
*Id.*

100 Romanis (2020), *supra* note 2.

101 Adkins (2024), *supra* note 17.

102 Romanis (2020), *supra* note 2, at 114.

103
*Paton, supra* note 86, per Sir George Baker at [279]; *Attorney-General’s Reference, supra* note 91—for an academic exploration of the distinction legally between birth and being born alive see: Romanis (2020), *supra* note 2.

104 Births and Deaths Registration Act 1953 s41(1).

105 ‘Mother’ is used here are a legal term of art—the law in England and Wales states that the person who gestates an infant will be legally recognized as ‘mother’ regardless of their intention or gender (see: *R (McConnell and YY) v Registrar General* [2020] 2 All ER 813, per Burnett LCJ, King LJ and Singh LJ at [89]).

106 M-BRRACE UK, *National Clinical Guidance for the Assessment of Signs of Life for Spontaneous Births Before 24 + 0 Weeks of Gestational Age where Active Survival-Focused Care is Not Appropriate 2020*, https://www.rcm.org.uk/media/4564/signs-of-life-guidance-document.pdf (accessed Jun. 10, 2023); Thomas Teague HHJ, *Chief Coroner’s Guidance No.45 Stillbirth, and Live Birth Following Termination of Pregnancy*, 12 (accessed Feb. 3, 2023).

107 Some have suggested that rather than a legal rule, this amounts to an evidentiary presumption in favor of life where the criteria are satisfied. See e.g.: Kristen Savell, *Is the Born Alive Rule Outdated and Indefensible*, 28 Syd. L. Rev. 625, 630 (2006) and Romanis (2020), *supra* note 2.

108 Jennifer Peterson, *Recognising Our ‘Invisible Infants’: There is no Internationally Agreed Definition of Live Birth—Is This Ethically Acceptable?*, 47 J. Med. Ethics 1, 2 (2021).

109 For example, RCOG Clinical Guidance outlines that ‘signs of life’ (eg, detectable heartbeat) are to be used in determining when intrauterine fetal death has occurred, clinically speaking. However, although signs of life play a role in ascertaining the death inside uterus in clinical terms, their role in assigning the legal status of stillbirth remains contingent upon the infant being outside of the gestating body: Royal College of Obstetricians and Gynaecologists, *Green Top Guideline, Guideline 55:Late Intrauterine Fetal Death and Stillbirth (October 2010)*, 2, https://www.rcog.org.uk/media/0fefdrk4/gtg_55.pdf (accessed Jun. 10, 2023).

110 M-BRRACE UK, *supra* note 106, at 5.

111
*Id.* at 5.

112 Romanis (2020), *supra* note 2, at 111. See also: Kate Greasley, Arguments About Abortion: Personhood, Mortality and Law 190 (2017).

113
*C v S* [1987] 1 All er 1230, per Donaldson mr at [149].

114 Romanis (2020), *supra* note 2.

115
*R v Iby* [2005] 63 Nswlr 278.

116
*Id.* at [56].

117
*R v Barratt (No 3)* [2019] Sasc 93, per Vanstone J at [112].

118 Romanis (2020d), *supra* note 78.

119 Romanis (2020), *supra* note 2.

120 Lucy Smith et al., *Parents’ Experiences of Care Following the Loss of a Baby at the Margins Between Miscarriage, Stillbirth and Neonatal Death: A UK Qualitative Study*, 127 Bjog: Int. J. Obstet. Gynaecol. 868, 869 (2020); Births and Deaths Registration Act 1926, s.12 (as amended).

121
*Id.*

122 Romanis (2020), *supra* note 2.

123 Colgrove, *supra* note 56.

124
*Id.* at 724.

125 Elselijn Kingma, *In Defence of Gestatelings: Response To Colgrove*’, 47 J. Med. Ethics 355 (2021); Romanis, *supra* note 2.

126 Romanis (2020b), *supra* note 15, at 206.

127 Lucy Smith et al., *Comparing Regional Infant Death Rates: The Influence of Preterm Births < 24 Weeks of Gestation*, 98 Arch. Dis. Child. Fetal. Neonatal. Ed. F103 (2013).

128 Louise Austin et al., *Effective Communication Following Pregnancy Loss: A Study in Engl*and, 30 Camb. Q. Healthc. 175 (2021).

129 Smith et al., *supra* note 120.

130 Danielle Fuller et al., *Preliminary Project Findings for Meeting with Representatives of the Department of Health and Social Care*, https://testprojectwebsiteblog.files.wordpress.com/2019/03/summary-of-project-findings-report-for-dhsc.pdf (accessed Sept. 12, 2023); Middlemiss and Kilshaw, *supra* note 67.

131 Erica van der Sijpt, *Navigating reproductive losses*, in The Routledge Handbook of Anthropology and Reproduction 351–364 (Sallie Han and Cecília Tomori eds., 2021); Karolina Kuberska and Sarah Turner, *The Presence of Absence: Tensions and Frictions of Pregnancy Losses–An Introduction*, 74 Women’s Stud. Int. Forum. 91 (2019).

132 Austin et al. (2021), *supra* note 128.

133 There are further questions to be explored about whether existing parental / bereavement leave arrangements would be an appropriate fit in the context of AAPT; however, a discussion of these issues falls out with the scope of this paper. For further consideration of these issues, see for example: Victoria Hooton and Chloe Romanis, *Artificial Womb Technology, Pregnancy, and EU Employment Rights*, 9 J. Law Biosc 1 (2022); Anna Nelson, *Neonatal Care (Leave and Pay) Act 2023: Some Questions Raised by Artificial Amniotic and Placenta Technology*, https://blog.petrieflom.law.harvard.edu/2023/09/11/neonatal-care-leave-and-pay-act-2023-some-questions-raised-by-artificial-amniotic-and-placenta-technology/ (accessed April 1, 2024)

134 Melvin Kranzberg, *Technology and History: ‘Kranzberg’s Laws.’,* 15 Bull. Sci. Technol. Soc. 5, 6 (1995).

135 Department of Health and Social Care, *Government Response to the Independent Pregnancy Loss Review,*https://www.gov.uk/government/publications/government-response-to-the-independent-pregnancy-loss-review (accessed Sept. 11, 2023).

